# Reduced exercise capacity in patients with systemic sclerosis is associated with lower peak tissue oxygen extraction: a cardiovascular magnetic resonance-augmented cardiopulmonary exercise study

**DOI:** 10.1186/s12968-021-00817-1

**Published:** 2021-10-28

**Authors:** James T. Brown, Tushar Kotecha, Jennifer A. Steeden, Marianna Fontana, Christopher P. Denton, J. Gerry Coghlan, Daniel S. Knight, Vivek Muthurangu

**Affiliations:** 1 Institute of Cardiovascular Science, University College London, London, UK; 2 Royal Free Hospital, London, UK; 3 Division of Medicine, University College London, London, UK; 4 Centre for Cardiovascular Imaging, Great Ormond Street Hospital for Children, Great Ormond Street, London, WC1N 3JH UK

**Keywords:** Exercise, Cardiopulmonary exercise testing, Cardiovascular magnetic resonance, Pulmonary arterial hypertension, Connective tissue disease, Systemic sclerosis

## Abstract

**Background:**

Exercise intolerance in systemic sclerosis (SSc) is typically attributed to cardiopulmonary limitations. However, problems with skeletal muscle oxygen extraction have not been fully investigated. This study used cardiovascular magnetic resonance (CMR)-augmented cardiopulmonary exercise testing (CMR-CPET) to simultaneously measure oxygen consumption and cardiac output. This allowed calculation of arteriovenous oxygen content gradient, a recognized marker of oxygen extraction. We performed CMR-CPET in 4 groups: systemic sclerosis (SSc); systemic sclerosis-associated pulmonary arterial hypertension (SSc-PAH); non-connective tissue disease pulmonary hypertension (NC-PAH); and healthy controls.

**Methods:**

We performed CMR-CPET in 60 subjects (15 in each group) using a supine ergometer following a ramped exercise protocol until exhaustion. Values for oxygen consumption, cardiac output and oxygen content gradient, as well as ventricular volumes, were obtained at rest and peak-exercise for all subjects. In addition, T1 and T2 maps were acquired at rest, and the most recent clinical measures (hemoglobin, lung function, 6-min walk, cardiac and catheterization) were collected.

**Results:**

All patient groups had reduced peak oxygen consumption compared to healthy controls (p < 0.022). The SSc and SSc-PAH groups had reduced peak oxygen content gradient compared to healthy controls (p < 0.03). Conversely, the SSc-PAH and NC-PH patients had reduced peak cardiac output compared to healthy controls and SSc patients (p < 0.006). Higher hemoglobin was associated with higher peak oxygen content gradient (p = 0.025) and higher myocardial T1 was associated with lower peak stroke volume (p = 0.011).

**Conclusions:**

Reduced peak oxygen consumption in SSc patients is predominantly driven by reduced oxygen content gradient and in SSc-PAH patients this was amplified by reduced peak cardiac output.

*Trial registration* The study is registered with ClinicalTrials.gov Protocol Registration and Results System (ClinicalTrials.gov ID: 100358).

**Supplementary Information:**

The online version contains supplementary material available at 10.1186/s12968-021-00817-1.

## Background

Exercise intolerance is common in systemic sclerosis (SSc), due to lung disease, myocardial involvement, pulmonary artery hypertension (PAH) or anemia. A relatively underinvestigated cause of reduced exercise capacity in these patients is skeletal muscle dysfunction. It has been shown that SSc patients have skeletal muscle inflammation, fibrosis and vasculopathy [1], all of which can reduce tissue oxygen extraction. Reduced oxygen extraction leads to reduced energy production, skeletal muscle dysfunction and ultimately exercise intolerance.

Understanding the relative importance of these factors is vital for targeting therapy. Unfortunately, this is difficult with conventional cardiopulmonary exercise testing (CPET) as only oxygen consumption (VO_2_) is directly measured. We have recently developed a novel technique that combines exercise cardiovascular magnetic resonance (CMR) with conventional CPET [2]. Our method (CMR-CPET) provides quantitative assessment of exercise capacity through combined direct measurement of both VO_2_ and cardiac output. The CMR measurements of cardiac output are considered gold-standard, eliminating some of the assumptions made by other non-invasive methods (e.g., Doppler echocardiography). Importantly, combining VO_2_ and cardiac output allows calculation of arteriovenous oxygen content gradient (∆avO_2_), a robust marker of tissue oxygen extraction [3]. CMR-CPET also enables accurate evaluation of ventricular function during exercise, providing a reference standard measure of contractile reserve. Finally, T1 and T2 mapping can also be performed in order to assess myocardial fibrosis and inflammation, which may be particularly pertinent in SSc patients.

The aim of this study was to use CMR-CPET to comprehensively investigate exercise capacity in patients with SSc. To achieve this, we investigated SSc patients with and without PAH. Furthermore, we included both a healthy control group and a disease control group consisting of patients with PAH not due to connective tissue disease (NC-PAH). The specific aims of this study were (1) To compare CMR-CPET metrics in the 4 groups, (2) correlate CMR-CPET metrics to clinical characteristics (6-min walk distance, lung function tests and hemoglobin) and (3) compare CMR-CPET metrics to myocardial T1 and T2 to identify associations with myocardial fibrosis/inflammation.

## Methods

### Study population

Sixty subjects were recruited between March 2019 and January 2021 with 15 subjects in each category—SSc, SSc-associated PAH (SSc-PAH), non-connective tissue disease pulmonary hypertension (NC-PAH: either idiopathic pulmonary arterial hypertension—IPAH, or chronic thromboembolic pulmonary arterial hypertension—CTEPH) and healthy controls. Patients were recruited from specialist clinics at our tertiary referral centre for connective tissue disease (CTD) and PAH. Inclusion criteria were (1) confirmed clinical diagnosis for patient groups and (2) age 18–80 years. In SSc patients, PAH was excluded by either right heart catheterization (5/15 patients) or by clinical evaluation and risk assessment with a validated risk-assessment tool [4] including, where necessary, echocardiography (10/15 patients). In the 2 PAH groups, PAH was diagnosed by right heart catheterization. Exclusion criteria were (1) general contraindications to CMR scanning, (2) contraindications to performing exercise test (unstable symptoms, including angina, exertional syncope, WHO class IV symptoms, and musculoskeletal disease preventing exercise), (3) previous symptomatic ischemic heart disease or moderate to severe valvular disease, (4) changes in targeted PAH therapy within 3 months and (5) significant lung parenchymal disease that may confound CPET results, such as interstitial lung disease (significant being defined as > 20% lung volume on computed tomography).

Clinical measures from the last outpatient appointment (including 6-min walk test in PAH groups) and the most recent lung function test data were collected in all patient groups. In addition, the most recent cardiac catheterization data were also collected in the 2 PAH groups.

The study was approved by national ethics committee (IRAS project ID 226101; REC reference 17/LO/1499, National Health Service Health Research Authority UK CRN 058274). All subjects provided written informed consent. The study is registered with ClinicalTrials.gov Protocol Registration and Results System (ClinicalTrials.gov ID: 100358).

### CMR-augmented cardiopulmonary exercise testing

Imaging was performed on a 1.5T CMR scanner (Magnetom Aera, Siemens Healthineers, Erlangen, Germany) using two 6-element coils (one spinal matrix, one body matrix). The scanning room was temperature controlled. Full resuscitation facilities were available. Each subject’s electrocardiogram (ECG) was monitored continuously using the in-built system in the CMR scanner. This system allowed assessment of rate and rhythm but is not suitable for identification of ischemia. All patients had peripheral venous access during testing for use in resuscitation protocols in the event of clinical instability.

### CMR imaging techniques (real-time flow and volume imaging)

Before exercise, subjects underwent a routine CMR with long- and short-axis cine imaging, myocardial native T1 and T2 mapping as previously described [5, 6].

**Fig. 1:**
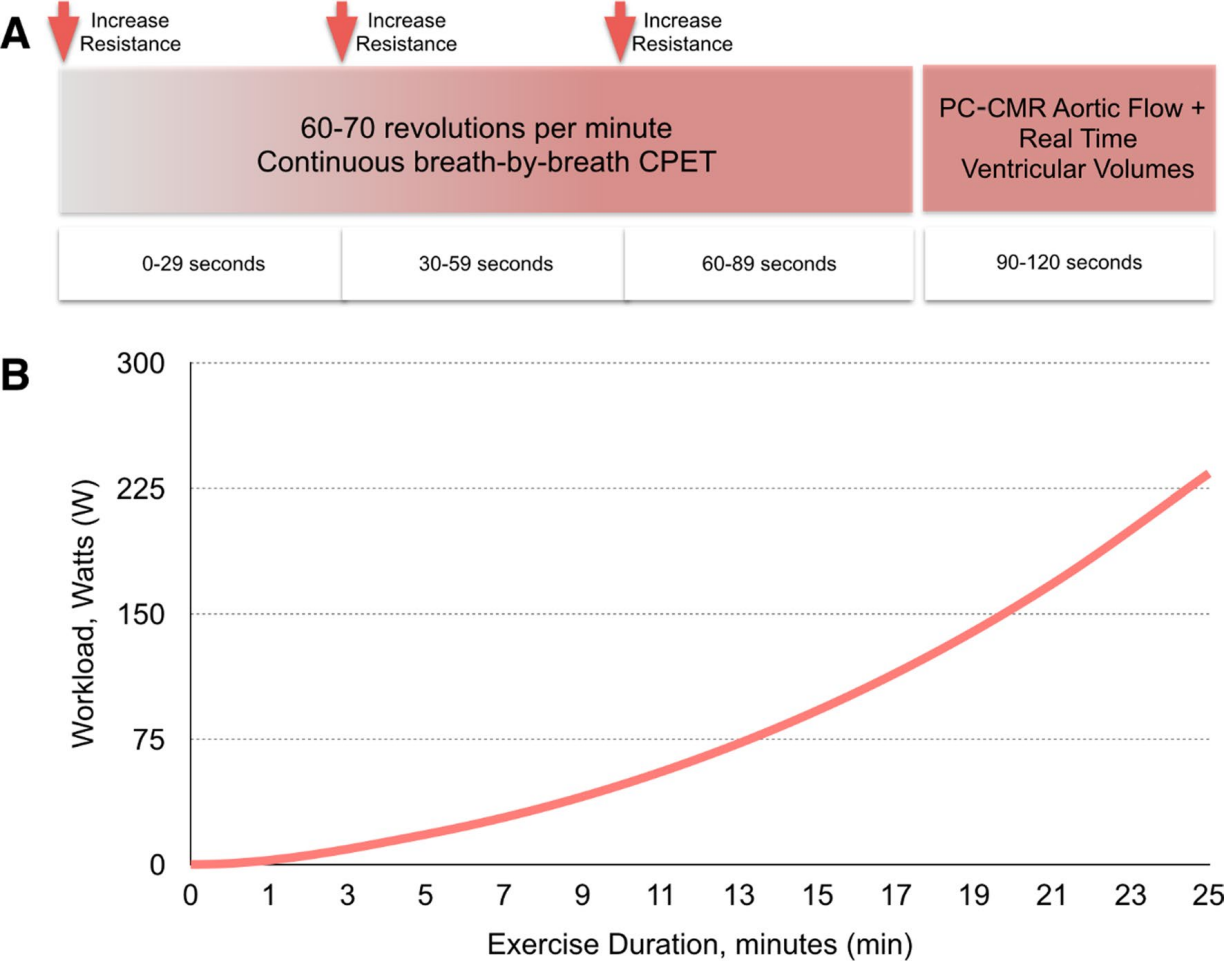
Exercise Protocol. **A** Timing of ergometer workload increases and imaging during each 2-min stage. **B** Cumulative workload against exercise duration for exercise protocol

Aortic flow was measured using real-time phase-contrast (PC) CMR (PC-CMR) at specified intervals during exercise (see Fig. 1A). PC-CMR was performed using a uniform density golden-angle spiral sequence, with a compressive sensing (CS) reconstruction [7]. The following parameters were used: matrix = 192 × 192, slice thickness = 7 mm, TR/TE = 9.8/1.6 ms, flip angle = 25°, velocity encoding = 300 cm/s, temporal resolution = ~ 41 ms, spatial resolution = 2.3 × 2.3 mm, acceleration factor = 6.

Real-time assessment of left ventricular (LV) and right ventricular (RV) volumes was performed immediately after each real-time flow acquisition using a 2D multi-slice real-time tiny golden-angle spiral CS balanced steady state free precession (bSSFP) sequence [8]. The following parameters were used: matrix = 208 × 208, slice thickness = 8 mm, TR/TE = 3.4/0.7 ms, flip angle = 67°, temporal resolution = ~ 31 ms, spatial resolution = 1.7 × 1.7 mm, acceleration factor = 8. Fourteen slices were used in each acquisition, with each slice being acquired over 2 R-R intervals. All real-time imaging was acquired during free breathing.

The aortic flow and short-axis image data were reconstructed off-line (MATLAB R2018a, MathWorks Inc, Natick, Massachusetts, USA), using the Berkeley Advanced Reconstruction Toolbox (BART) [9]. The reconstructed images were exported as DICOM files and analyzed on reporting workstations. 

### Respiratory gas analysis

Breath-by-breath gas exchange analysis was performed using a commercial CPET system (Ultima, MedGraphics, St Paul, Minnesota, USA). The analyzer was placed in the CMR control room and attached to the facemask (Hans Rudolph, Kansas City, Kansas, USA) via a set of CMR-compatible sampling tubes (umbilicus) passed through the waveguide. This bespoke umbilicus was modified as previously described [2] increasing overall length from the standard 234–1000 cm and removing ferromagnetic components. It was thoroughly tested by the manufacturer, meeting all quality control standards. Gas and flow calibrations were performed before each test and at least 30 min after system initiation. All measurements were taken at body temperature and ambient pressure.

### Exercise protocol

Subjects performed exercise on a supine CMR-compatible cycle ergometer (MR Cardiac Ergometer Pedal, Lode, Groningen, Netherlands). This ergometer allowed exercise workload (power measured in Watts—W) to be controlled by altering resistance depending on cadence. Subjects were briefed before their scan and familiarized with the equipment and protocol. Baseline aortic flow and ventricular volumes are acquired before commencing the ramped exercise protocol.

The first minute consisted of exercise against zero resistance, with subjects asked to cycle at 60–70 rpm. Thereafter, the protocol was split in 2-min stages. During each stage, workload was increased at 0, 30 and 60 s with acquisition of aortic flow and ventricular volumes commenced at 90 s. Workload increments at 0, 30 and 60 s varied by stage as follows—stages 1–3: 3 W, stages 4–6: 5 W, stage 7–8: 7 W, stages 9–10: 9 W and stages 11–12: 11 W (Fig. 1B). The smaller increments at the start of the protocol ensured that even subjects with significant exercise intolerance were able to complete at least 2 exercise stages.

This protocol was followed until exhaustion and at the onset of exhaustion (defined as an inability to maintain cadence or a verbal indication from the subject) the subject was encouraged to maintain cycling while peak aortic flow and ventricular volumes were acquired. Exercise was then stopped, followed by a 15-min recovery period with monitoring of vital signs in the CMR room.

### Data analysis

All post-processing of the reconstructed images was performed using ‘in-house’ plug-ins for the open-source OsiriX DICOM software version 9.0.1 (OsiriX Foundation, Geneva, Switzerland) [10–12].

PC-CMR flow data of the ascending thoracic aorta was segmented using a semi-automatic vessel edge detection algorithm with manual operator correction if required. Stroke volume (SV) was calculated by integrating the flow curve across a single R-R interval. Cardiac output was given by SV x heart rate (HR). LV and RV endocardial borders were traced manually on the short-axis images at end-diastole and end-systole, identified by visual assessment of the largest and smallest cavity areas, respectively. Papillary muscle and trabeculae were excluded from the blood pool. Biventricular stroke volumes were calculated as the difference between the end-diastolic volume (EDV) and end-systolic volume (ESV), and ejection fraction (EF) was determined as (SV/EDV) × 100. All measurements were reported by an experienced clinical CMR specialist (DK) blinded to the clinical information. All volumetric data and cardiac output were indexed to body surface area (BSA) and denoted by the suffix − i.

VO_2_ and respiratory exchange ratio (RER) measurements were time registered to CMR data. The VO_2_ was indexed to body weight and denoted by the prefix − i. Arteriovenous oxygen content gradient was calculated as ∆avO_2_ = VO_2_/CO (using non indexed data). These calculations were performed at rest and peak-exercise for all subjects.

### Statistical analysis

All statistical analyses were performed using R (version 3.2.0, The R Foundation for Statistical Computing, Vienna, Austria). Data were examined for normality using the Shapiro–Wilk normality test. Descriptive statistics were expressed as mean (± standard deviation) for normally distributed data and median (range) for non-normally distributed data. Main effects of disease type and exercise and an interaction term representing disease multiplied by exercise for CMR-CPET metrics were assessed using repeated measures ANOVA (normal data) and aligned rank repeated measures ANOVA (non-normal data). Between-group differences (at rest and exercise) were assessed using 1-way analysis of variance (ANOVA) for normal data and the Kruskill Wallis for non-normal data. Post hoc comparisons were performed using pairwise t-tests (normal data) and Mann Whitney tests (non-normal data) with Benjamini Hochberg correction for multiple comparisons. Sex distribution between the groups was assessed using the Chi-squared test. Correlation between metrics corrected for diagnosis was computed using multi-level Spearman’s rank partial correlation coefficient. A *p* value < 0.05 was considered statistically significant.

## Results

### Demographics and clinical data

There were no significant differences in age, sex, height, weight or BSA between the groups (Table 1). In both the SSc and SSc-PAH groups, 14 patients (93%) had limited cutaneous systemic sclerosis, while the remaining patient had a diagnosis of diffuse cutaneous systemic sclerosis (Auto-antibody specificities detailed in Additional file 1: Table S1). In the NC-PAH group, 10 (67%) patients had IPAH and 5 (33%) had CTEPH.

**Table 1:** Subject characteristics

	SSc	SSc-PAH	NC-PAH	Healthy Controls	*p* value
Age (years)	52.5 ± 14.0	59.7 ± 8.4	51.1 ± 18.1	51.3 ± 4.2	0.19
Height* (m)	1.6 (1.6–1.8)	1.6 (1.5–1.9)	1.6 (1.5–1.9)	1.6 (1.6–1.8)	0.95
Weight (kg)	66.2 ± 14.5	68.6 ± 14.3	75.7 ± 18.4	70.4 ± 13.0	0.38
BSA (m^2^)	1.7 ± 0.2	1.8 ± 0.2	1.8 ± 0.2	1.8 ± 0.2	0.72
M/F	2/13	3/12	7/8	3/12	0.15

*BSA* body surface area, *SSc* systemic sclerosis, *SSc-PAH* systemic sclerosis-associated pulmonary arterial hypertension, *NC-PAH* non-connective tissue disease pulmonary artery hypertension. Normally distributed data displayed as mean ± SD. *Denotes non-normally distributed, data shown as median (range)

Clinical characteristics (including hemoglobin) from the most recent clinic visit (32 days, interquartile range 6–60 days) and lung function test data [199 days, interquartile range (IQR) 13–425 days] are shown in Table 2. The most recent cardiac catheterization (218 days, IQR 57–839 days) and 6 min walk test (6MWT) (32 days, IQR 6–60 days) data from the 2 PAH groups are also shown in Table 2.

**Table 2:** Hemodynamic measurements for pulmonary hypertension groups, clinical measurements for all patient groups

	SSc	SSc-PAH	NC-PAH	*p* value
Hemoglobin (g/L)	121.9 ± 11.4	123.5 ± 14.2	133.3 ± 22.2	0.14
FEV1 (%predicted)	95.5 ± 17.5	85.5 ± 13.2	88.9 ± 14.7	0.20
FVC (%predicted)	98.0 ± 19.8	91.8 ± 15.6	98.1 ± 18.3	0.56
DLCO (%predicted)	67.4 ± 14.7	39.4 ± 14.1^‡§^	64.3 ± 16.9	< 0.001
PVR* (dynes/s/cm^−5^)	–	346 (146–1373)	444 (180–1358)	0.17
mPAP* (mmHg)	–	32 (20–80)	39 (25–64)	0.17
6MWT (m)	–	372 ± 105	451 ± 94	0.042

*PVR* pulmonary vascular resistance, *mPAP* mean pulmonary arterial pressure, *6MWT* 6-minute walk test, *FEV1* forced expiratory volume in 1 s, *FVC* forced vital capacity, *DLCO* diffusion capacity of the lung for carbon monoxide. Normally distributed data displayed as mean ± SD. *Denotes non-normally distributed data shown as median (range). ^‡^Significant difference between SSc and SSc-PAH groups. ^§^Significant difference between NC-PAH and the indicated SSc groups

The main findings were that (1) 6MWT was significantly shorter in the SSc-PAH group compared to NC-PAH group (*p* = 0.042) and (2) the measured diffuse lung capacity (DLCO) as a percentage of the predicted value was significantly lower in the SSc-PAH group compared to the other 2 patient groups (*p* < 0.001). There were no other significant differences in clinical characteristics (including predicted forced expiratory volume in one second (FEV1), predicted forced vital capacity (FVC), and Hb). Medications for all patients are shown in Additional file 1: Table S1.

### Resting CMR-CPET

Resting CMR metrics for each of the groups are shown in Table 3. The main difference in functional metrics was a significantly higher cardiac index in the patient groups compared to healthy controls (*p* ≤ 0.023). In SSc patients this was associated with higher stroke volume index (*p* = 0.017) compared to healthy controls whilst in NC-PAH patients, higher HR was observed compared to healthy controls (*p* = 0.005). There were no group differences in biventricular function.

**Table 3:** Resting CPET and CMR metrics

	SSc	SSc-PAH	NC-PAH	Healthy Controls	*p* value
iVO_2_* (ml/min/kg)	3.3 (2.6–4.7)	3.4 (2.9–5.0)	3.1 (1.7–5.3)	3.1 (2.6–4.2)	0.14
Cardiac index (L/min/m^2^)	3.1 ± 0.7^†^	3.0 ± 0.8^†^	3.0 ± 0.7^†^	2.3 ± 0.4	0.008
avO_2_*(mlO_2_/100 ml)	4.3 (2.3–6.7)^†^	4.3 (3.3—9.2)	4.4 (3.0–6.6)^†^	5.4 (4.0–7.2)	0.007
SV index (ml/m^2^)	45.5 ± 7.7^†§^	41.4 ± 9.7	37.3 ± 10.1	35.4 ± 7.5	0.014
HR (bpm)	69.5 ± 9.1^§^	72.5 ± 9.0	80.6 ± 10.1^†^	67.0 ± 13.4	0.005
RVEDVI (ml/m^2^)	70.2 ± 12.1	71.7 ± 13.9	69.7 ± 14.5	61.4 ± 16.2	0.21
RVESVI* (ml/m^2^)	24.5 (10.5–40.9)	27.7 (19.0–47.8)	29.1 (12.7–64.8)	23.6 (14.9–58.9)	0.20
RVSVI (ml/m^2^)	44.0 ± 7.2^†§^	40.2 ± 9.4	34.9 ± 9.0	34.8 ± 7.2	0.008
LVEDVI (ml/m^2^)	67.7 ± 11.4^§^	63.7 ± 12.9	54.7 ± 16.3	58.0 ± 9.9	0.037
LVESVI (ml/m^2^)	23.7 ± 8.3	22.9 ± 7.6	19.6 ± 10.5	22.8 ± 5.9	0.54
LVSVI (ml/m^2^)	44.1 ± 7.2^†§^	40.8 ± 9.7	35.1 ± 8.8	35.3 ± 6.7	0.008
LVEF (%)	66 ± 8	64 ± 9	66 ± 10	61 ± 7	0.38
RVEF* (%)	64 (53–81)	59 (30–66)	56 (22–73)	59 (42–67)	0.10
T2* (ms)	51 (44–57)^†§^	50 (47–62)^†§^	48 (43–52)	46 (42–49)	< 0.001
T1 (ms)	1076 ± 44^†^	1079 ± 50^†§^	1043 ± 46^†^	994 ± 23	< 0.001

*iVO*_*2*_ oxygen consumption indexed to weight, *BSA*, body surface, *avO*_*2*_ tissue oxygen extraction, *SV* stoke volume, *HR* heart rate, *RVEDVI* BSA indexed right ventricular end diastolic volume, *RVESVI* BSA indexed right ventricular end systolic volume, *RVSVI* BSA indexed right ventricular stroke volume, *RVEF* right ventricular ejection fraction, *LVEDVI/LVESVI/LVSVI/LVEF* left ventricular measurements as per RV. Normally distributed data displayed as mean ± SD. *Denotes non-normally distributed data shown as median (range). ^†^Significant difference between controls and indicated patient groups. §significant difference between NC-PAH and the indicated SSc groups

Myocardial T2 was higher in the SSc and SSc-PAH groups compared to both controls and NC-PAH patients (*p* < 0.006). Myocardial T1 was higher in all patient groups compared to healthy controls (*p* < 0.006) and was higher in SSc-PAH compared to NC-PAH patients (*p* = 0.042).

There were no group differences in resting iVO_2_ (Table 3) but resting ∆avO_2_ was significantly lower in the SSc patients (*p* < 0.001) and NC-PAH patients (*p* = 0.049) compared to healthy controls.

### Exercise feasibility

All subjects successfully completed the exercise protocol and no subjects required medical intervention. The peak-exercise RER ≥ 1.0 in 59/60 patients, with 1 SSc-PAH patient, who exercised to exhaustion, achieving an RER of 0.97. Comparisons of exercise duration and peak workload are shown in Table 4.

**Table 4:** Exercise metrics for all groups

Variable	SSc	SSc-PAH	NC-PAH	Healthy Controls	*p* value
Exercise duration (s)	706 ± 150^†^	503 ± 166^†‡^	592 ± 256^†^	913 ± 158	< 0.001
Peak workload* (W)	62 (27–107)^†^	37 (21–107)^†‡^	52 (18–141)^†^	100 (62–168)	< 0.001
Maximum RER*	1.4 (1.1–1.6)^†§^	1.2 (1.0–1.4)^†‡^	1.2 (1.0–1.6)^†^	1.5 (1.1–2.1)	< 0.001

*RER* respiratory exchange ratio. Normally distributed data displayed as mean ± SD. *Denotes non-normally distributed data shown as median (range). ^†^Significant difference between controls and indicated patient groups. ^‡^Significant difference between SSc and SSc-PAH groups. ^§^significant difference between NC-PAH and the indicated SSc groups

The SSc-PAH group had the lowest peak workload and shortest exercise duration (significantly different from controls and SSc patients—*p* < 0.003 for peak workload, *p* < 0.006 for exercise duration). Peak workload was also lower in NC-PAH patients compared to controls (*p* < 0.002).

### Exercise CMR-CPET metrics

**Fig. 2:**
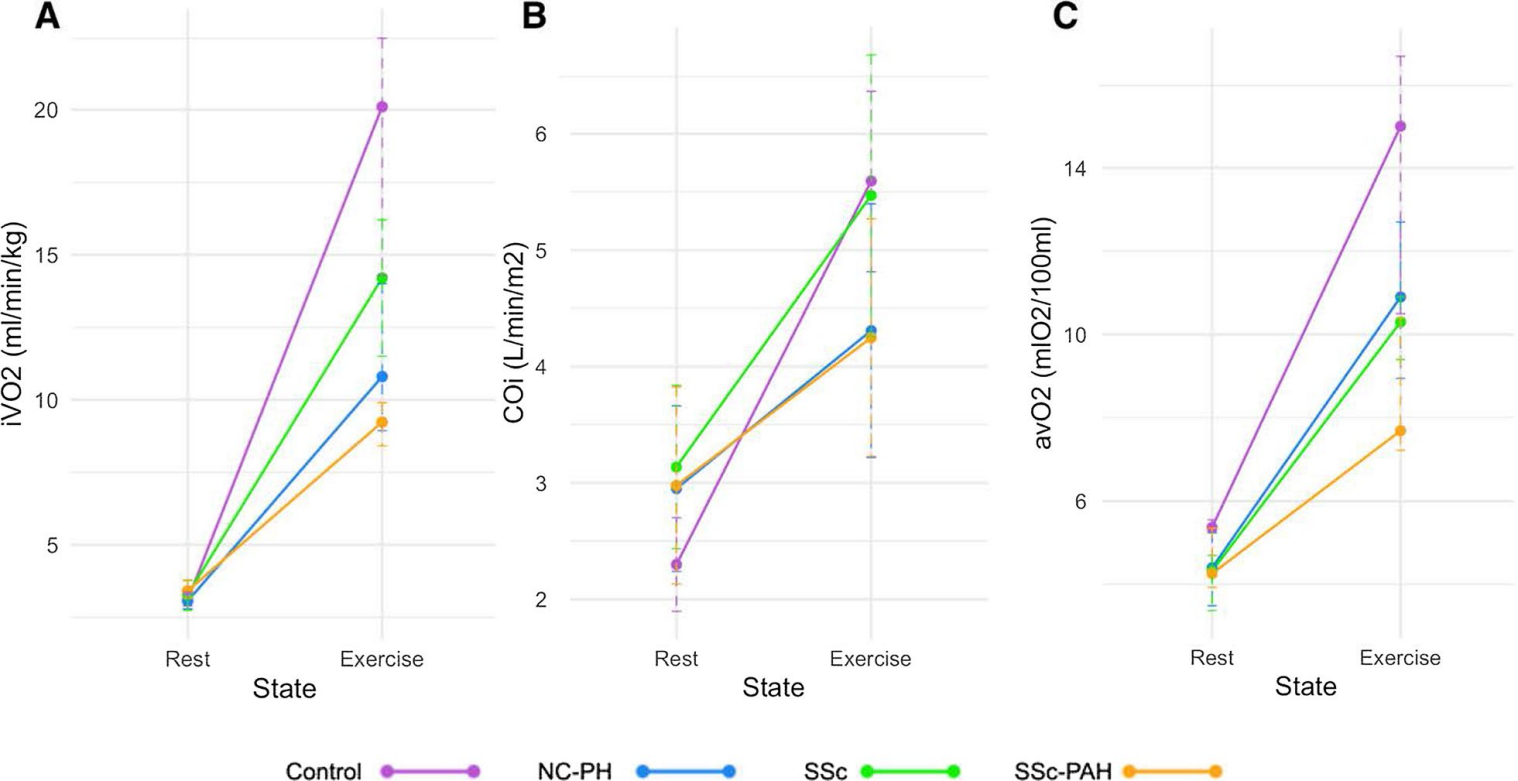
CMR-CPET Metrics at rest and peak-exercise for each subject group: Systemic Sclerosis (SSc); Systemic Sclerosis-associated Pulmonary Arterial Hypertension (SSc-PAH); Non-Connective Tissue Disease Pulmonary artery Hypertension (NC-PAH); Healthy controls. **A** Oxygen consumption indexed to weight (iVO_2_)*. **B** Cardiac output indexed to body surface area. **C** Arteriovenous oxygen gradient (∆avO_2_)*. Normally distributed data displayed as mean ± SD. *Denotes non-normally distributed data shown as median (range)

CMR-CPET metrics at peak exercise are shown in Table 5. All patient groups had significantly lower peak iVO_2_ than healthy controls (*p* < 0.022) as seen in Fig. 2.

**Table 5:** Peak exercise CPET and CMR metrics

	SSc	SSc-PAH	NC-PAH	Healthy Controls	*p* value
iVO_2_* (ml/min/kg)	14.2 (7.6–26.2)^†^	9.2 (5.2–15.7)^†‡^	10.8 (6.6–19.9) ^†^	20.1 (12.8–28.7)	< 0.001
Cardiac index (L/min/m^2^)	5.5 ± 1.2^§^	4.2 ± 1.0^†‡^	4.3 ± 1.1^†^	5.6 ± 0.8	< 0.001
avO_2_* (mlO_2_/100 ml)	10.3 (4.6–11.8) ^†^	7.7 (6.1–12.9)^†^	10.9 (6.9–17.5)	15.0 (7.1–18.7)	0.003
SV index (ml/m^2^)	44.8 ± 10.2	41.6 ± 8.2	37.0 ± 9.7^†^	47.1 ± 10.2	0.034
HR (bpm)	124.0 ± 16.5	102.7 ± 14.0^†‡§^	118.4 ± 22.4	122.1 ± 18.8	0.009
LVEF (%)	72 ± 12	76 ± 8	78 ± 10	77 ± 7	0.24
RVEF* (%)	71 (43–84) ^§^	66 (42–79) ^†^	59 (24–81) ^†^	74 (62–89)	0.003

*iVO*_*2*_ oxygen consumption indexed to weight, *avO*_*2*_ tissue oxygen extraction, *HR* heart rate, *LVEF* left ventricular ejection fraction, *RVEF* right ventricular ejection fraction. Normally distributed data displayed as mean ± SD. *Denotes non-normally distributed data shown as median (range). ^†^Significant difference between controls and indicated patient groups. ^‡^Significant difference between SSc and SSc-PAH groups. §significant difference between NC-PAH and the indicated SSc groups

**Fig. 3:**
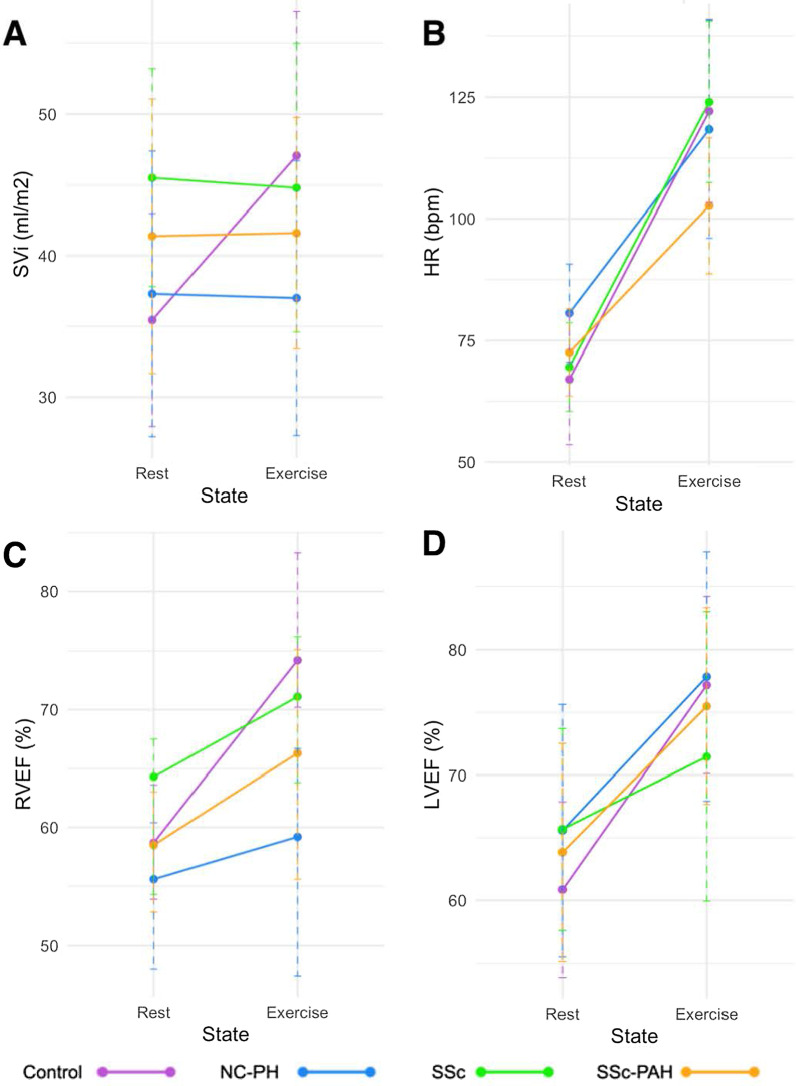
Stroke volume, heart rate and ventricular ejection fraction at rest and peak-exercise for each subject group: Systemic Sclerosis (SSc); Systemic Sclerosis-associated Pulmonary Arterial Hypertension (SSc-PAH); Non-Connective Tissue Disease Pulmonary Hypertension (NC-PAH); Healthy controls. **A** Stroke volume indexed to body surface area. **B** Heart rate (HR). **C** Right ventricular ejection fraction (RVEF)*. **D** Left ventricular ejection fraction (LVEF). Normally distributed data displayed as mean ± SD. *Denotes non-normally distributed data shown as median (range)

The SSc-PAH group had the lowest peak iVO_2_ (significantly different from both healthy controls and SSc patients—*p* < 0.01). This was associated with lower peak cardiac index compared to both healthy controls and SSc patients (*p* ≤ 0.004) and lower ∆avO_2_ than healthy controls (*p* = 0.003)—Fig. 2. The lower peak cardiac index during exercise was due to a failure to augment SV and lower peak HR compared to healthy controls (*p* = 0.015)—Fig. 3. The SSc-PAH group also had a lower peak RVEF compared to healthy controls (*p* = 0.042)—Fig. 3.

SSc patients had lower peak iVO_2_ and ∆avO_2_ (*p* = 0.03) compared to healthy controls. However, they did have significantly higher peak iVO_2_ than SSc-PAH patients (*p* = 0.01). SSc patients failed to augment stroke inde during exercise (Fig. 2) but peak stroke index was similar to healthy controls, due to higher resting stroke index. This also resulted in a similar peak cardiac index compared to healthy controls. All other peak functional metrics were not statistically different from healthy controls.

The NC-PAH group was characterized by significantly lower peak cardiac index compared to both healthy controls and SSc patients (*p* ≤ 0.005)—Fig. 2. The NC-PAH group also had the lowest peak stroke index (significantly different to healthy controls—*p* = 0.034) and RVEF (statistically significant versus healthy controls and SSc patients—*p* ≤ 0.042)—Fig. 3.

Peak iVO_2_, cardiac index and ∆avO_2_ all correlated with 6MWT after adjusting for diagnosis (*p* ≤ 0.043). There was no correlation between 6MWT and peak RVEF, LVEF, stroke index or HR.

### Relationship between exercise metrics and lung function and Hemoglobin

There was no correlation between predicted FVC and FEV1 and any peak-exercise metrics. There was a correlation (adjusted for diagnosis) between DLCO and peak RVEF and LVEF (peak RVEF rho = 0.37, *p* = 0.015; peak LVEF rho = 0.31, *p* = 0.040). In addition, there was a significant correlation (adjusted for diagnosis) between peak ∆avO_2_ and Hb (rho = 0.33, *p* = 0.025).

### Relationship between exercise metrics and myocardial T1 and T2

There were no significant associations between CMR-CPET and myocardial T2 after adjusting for diagnosis. Myocardial T1 correlated (adjusted for diagnosis) with peak stroke volume index (rho = − 0.33, *p* = 0.011) and HR (rho = 0.31, *p* = 0.020). There were no other significant associations.

## Discussion

It is well recognized that systemic sclerosis causes reduced exercise capacity [13], which is often attributed to lung disease or PAH [14]. However, patients without lung disease also experience exercise intolerance, and PAH only affects a minority of patients with SSc (8–12% prevalence) [15]. Thus, other factors must be important, and we used CMR-CPET to better determine the causes of exercise intolerance in SSc. To investigate the separate contributions of SSc and PAH to exercise intolerance, we compared SSc patients (with and without PAH) to healthy controls and NC-PAH patients. The main findings of the study were: (1) All patient groups had reduced peak iVO_2_ compared to healthy controls, (3) SSc and SSc-PAH had reduced peak ∆avO_2_ compared to healthy controls and NC-PAH patients (2) SSc-PAH and NC-PAH patients had reduced peak cardiac index compared to healthy controls and SSc patients, (4) Higher hemoglobin was associated with higher peak ∆avO_2_ independent of disease type and (5) Higher myocardial T1 was associated with lower peak SV.

The defining exercise feature of SSc and SSc-PAH patients was reduced peak VO_2_, associated with lower peak ∆avO_2_ in both groups. It is well recognized that ∆avO_2_ is a marker of skeletal muscle oxygen extraction, and our data suggest that reduced oxygen extraction is a ubiquitous problem in SSc. The ability of skeletal muscle to extract oxygen is vital for normal aerobic respiration and thus cellular function. From our data, we propose the following in SSc patients during exercise—(1) reduced muscle oxygen extraction, (2) reduced aerobic respiration and ATP production, (3) reduced skeletal muscle sarcomeric contraction and (4) resultant exercise intolerance. This chain of events has previously been suggested for left-sided heart failure [16] and pediatric PAH [17] and we believe is equally important in SSc. The etiology of reduced peak ∆avO_2_ in these patients is probably generalized skeletal muscle dysfunction/sarcopenia. Sarcopenia is common in SSc [18], and disease specific causes include small vessel vasculopathy [1, 19], muscle fibrosis and inflammation [20]. These factors not only cause sarcopenia, but also directly limit oxygen extraction through reduced regional O_2_ delivery, intramuscular shunting and mitochondrial dysfunction [21]. In this study, we did not explore the exact nature of skeletal muscle involvement in SSc. However, this will be an important feature of future studies, particularly identifying causes amenable to therapeutic interventions.

Interestingly, we found a strong relationship between hemoglobin and peak ∆avO_2_. There were no group differences in hemoglobin and, therefore, this finding cannot explain the group differences in oxygen extraction but may contribute to within-group variance. Several animal studies have shown that low hemoglobin causes reduced oxygen extraction through impaired oxygen delivery. There is also some evidence that anemia lowers muscle oxidative capacity [22] and this may further contribute to lower oxygen extraction [23]. Anemia and iron deficiency are well described in chronic diseases such as PAH. However, iron supplementation has a limited effect on exercise capacity in these patients [24]. On the other hand, anemia in SSc may have a more inflammatory component and anti-inflammatory drugs have been shown to increase Hb [25–28], which might improve exercise capacity.

In addition to reduced peak ∆avO_2_, patients with SSc-PAH also had lower peak cardiac index. This ‘dual pathology’ probably explains why SSc-PAH patients had the lowest peak VO_2_ and a lower 6MWT than NC-PAH patients. Reduced peak cardiac index was also seen in NC-PAH patients, and in both groups this was largely due to an inability to augment stroke index during exercise. Interestingly, SSc patients also failed to augment stroke index, but higher resting stroke index resulted in normal peak values. Higher baseline stroke index in SSc patients may simply be a response to a systemic inflammatory disease, but this requires more investigation. The failure to augment stroke index can be explained by poor RV contractile reserve and this is reflected by lower peak RVEF in both PAH groups. Reduced contractile reserve is well recognized in PAH [29, 30] due to both RV dysfunction [31] and increased afterload [32]. In addition, autonomic failure and reduced inotropy maybe a factor, which is in keeping with the reduced peak HR seen in SSc-PAH patients. Interestingly, we also found that peak stroke index was associated with increased myocardial native T1 but not T2. This suggests that fibrotic changes in the myocardium (secondary to raised afterload and/or burnt-out myocarditis) may partly underlie the loss of contractile reserve.

Another possible cause of reduced ∆avO_2_ in SSc is lung disease, which is why we excluded patients with radiological evidence of extensive parenchymal lung disease. We did not find any association between lung function metrics and peak ∆avO_2_, implying that reduced pulmonary O_2_ uptake was not the cause of lower ∆avO_2_. However, there was an association between predicted DLCO and peak biventricular EF, but the direction of causation is unclear, and the exact cause is to be determined.

In this study, we used CMR-augmented CPET to investigate exercise dysfunction. The benefit of this relatively novel technology is that it allows simultaneous quantification of peak iVO_2_ and cardiac index and subsequent calculation of ∆avO_2_. We used PC-CMR to measure aortic flow as this provides accurate quantification of cardiac output even in the presence of left-sided valvar regurgitation and shunts. This contrasts with measurement of cardiac output from ventricular volumetric data. Aortic flow can also be estimated using Doppler echocardiography, but this method is often unreliable [33] and difficult to perform during exercise. In addition, CMR also provides reference standard ventricular volumetric and mapping data. Our findings demonstrate that CMR-CPET can help develop a better understanding of the causes of exercise dysfunction in different types of disease. In particular, quantification of ∆avO_2_ provides new insights into the role of skeletal muscle in reduced exercise capacity. Due to the comprehensive evaluation that CMR-CPET provides, we believe that this technique may have a future role in clinical diagnosis, risk stratification and follow-up, as well as providing end points for clinical trials.

### Limitations

The main limitation of this study is that achieving true peak-exercise using a supine exercise protocol is challenging. Our form of supine exercise is not directly comparable with conventional CPET, and this should be considered when interpreting the results. Nevertheless, we have previously demonstrated good correlation between peak iVO_2_ obtained during CMR-CPET and conventional CPET [2]. Furthermore, even though peak VO_2_ and HR during supine exercise are lower than for upright exercise, RER > 1 was achieved in most subjects. Therefore, we believe that CMR-CPET metrics measured during supine exercise remain good markers of exercise capacity.

Another limitation was the long time interval between clinical measures (i.e., lung function tests) and CMR-CPET. This may affect the robustness of correlations between these markers and future, larger studies should endeavor to assess clinical characteristics at the same time as CMR-CPET.

Finally, skeletal muscle biopsy could have answered some of our questions regarding the cause of reduced oxygen extraction (fibrosis/inflammation/capillary rarefaction). However, this is highly invasive and would have been difficult to perform. Thus, future studies could evaluate skeletal muscle mass, perfusion, T2 and extracellular volume as a non-invasive alternative.

## Conclusions

Patients with SSc and SSc-PAH have reduced peak iVO_2_. In SSc patients, this appears related to reduced peak ∆avO_2_; in SSc-PAH patients, it is related to both reduced peak cardiac index *and* ∆avO_2_. This suggests that tissue oxygen extraction is an important determinant of exercise intolerance in SSc and could be used as a biomarker of disease and response to therapy.

## Supplementary Information

**Additional file 1**. **Table S1.** Subject characteristics.

## Data Availability

The datasets used and analysed during the current study are available from the corresponding author on reasonable request.

## Abbreviations

∆avO2: Arteriovenous oxygen content gradient
6MWT: Six minute walk test
BSA: Body surface area
bSSFP: Balanced steady state free precession
CMR: Cardiovascular magnetic resonance
CPET: Cardiopulmonary exercise testing
CTD: Connective tissue disease
CTEPH: Chronic thromboembolic pulmonary hypertension
DLCO: Diffusion lung capacity for carbon monoxide
ECG: Electrocardiogram
EDV: End-diastolic volume
EF: Ejection fraction
ESV: End-systolic volume
FEV1: Forced expiratory volume in one second
FVC: Forced vital capacity
HR: Heart rate
IPAH: Idiopathic pulmonary artery hypertension
LV: Left ventricle/left ventricular
LVEDVI: Left ventricular end-diastolic volume index
LVEF: Left ventricular ejection fraction
LVESVI: Left ventricular end-systolic volume index
mPAP: Mean pulmonary artery pressure
NC: Non-connective tissue
PAH: Pulmonary artery hypertension
PC: Phase contrast
PVR: Pulmonary vascular resistance
RER: Respiratory exchange ratio
RV: Right ventricle/right ventricular
RVEDVI: Right ventricular end-diastolic volume index
RVEF: Right ventricular ejection fraction
RVESV: Right ventricular end-systolic volume
RVESVI: Right ventricular end-systolic volume index
SSc: Systemic sclerosis
SV: Stroke volume
SVI: Stroke volume index
VO2: Oxygen consumption
