# Supplementary material for: Reduced exercise capacity in patients with systemic sclerosis is associated with lower peak tissue oxygen extraction: a cardiovascular magnetic resonance-augmented cardiopulmonary exercise study
Source: J Cardiovasc Magn Reson. 2021 Oct 28;23:118. doi: 10.1186/s12968-021-00817-1 (PMC8554852; doi:10.1186/s12968-021-00817-1)
Supplement: Supplementary file 1 — Additional file 1. Table S1. Subject characteristics. [file 12968_2021_817_MOESM1_ESM.docx]

|  | SSc | SSc-PAH | NC-PH |
| --- | --- | --- | --- |
|  | | | |
| ACA | 7 | 13 | - |
| ANA | 1 | - | - |
| Anti-RNA polymerase III | 1 | - | - |
| Anti-RNP | 1 | 2 | - |
| Anti-Scl70 | 5 | - | - |
|  | | | |
| Phosphodiesterase-5 inhibitor | - | 13 | 11 |
| Endothelin receptor antagonist | - | 11 | 9 |
| Intravenous epoprostenol | - | 2 | 1 |
| Selective prostacyclin receptor antagonist | - | 1 | 1 |
| Soluble guanylate cyclase stimulator | - | 1 | 1 |

**Table 1. Subject characteristics.**

SSc = Systemic Sclerosis; SSc-PAH = Systemic Sclerosis-associated Pulmonary Arterial Hypertension; NC-PH = Non-Connective Tissue Disease Pulmonary Hypertension; ACA = Anti-Centromere Antibodies; ANA = Anti-Nuclear Antibodies; RNA = Ribonucleic Acid; RNP = Ribonucleoprotein.
